# The effect of emotional freedom technique on depressive and menopausal symptoms in postmenopausal women: randomized controlled trial

**DOI:** 10.1590/1806-9282.20260133

**Published:** 2026-08-24

**Authors:** Meltem Uğurlu, Derya Yuksel Koçak, Meryem Vural Şahin

**Affiliations:** 1 University of Health Sciences, Gulhane Faculty of Nursing, Department of Obstetrics and Gynecology Nursing – Ankara, Turkey.; 2 University of Health Sciences Turkey, Institute of Hitit Health Sciences, Department of Nursing – Çorum, Turkey.; 3 University of Health Sciences, Gulhane Faculty of Health Sciences, Department of Midwifery – Ankara, Turkey.

**Keywords:** Complementary therapy, Depression, Women's health, Menopause

## Abstract

**OBJECTIVE:**

The aim of this study was to investigate the effects of the Emotional Freedom Technique on depressive and menopausal symptoms among postmenopausal women.

**METHODS:**

The study sample consisted of 70 postmenopausal women (Emotional Freedom Technique group: 34; control group: 36). Participants in the intervention group received Emotional Freedom Technique at least once daily for 8 weeks. The control group received only routine care. Data were collected using a personal information form, the Menopause Rating Scale and the Beck Depression Inventory. All assessments were conducted before and after the intervention. Analyses were conducted using G*Power and Statistical Package for the Social Sciences 25.

**RESULTS:**

Emotional Freedom Technique significantly reduced the Beck Depression Inventory scores (p<0.001), as well as Menopause Rating Scale total (p<0.001) and subdimension scores: somatic (p<0.001) and psychological (p<0.001), and urogenital (p=0.005). Following the intervention, Beck Depression Inventory score decreased by 62.8%, Menopause Rating Scale total score by 46.3%, somatic subscale score by 52.1%, psychological subscale score by 45.0%, and urogenital subscale score by 20.4%.

**CONCLUSION:**

Emotional Freedom Technique shows promise as a potential adjunctive intervention, but further blinded, controlled trials are needed. The Emotional Freedom Technique was effective in reducing depression and menopausal symptoms among postmenopausal women after 8 weeks of daily practice. It is suggested that its use alongside standard care may help alleviate depressive and menopausal symptoms.

## INTRODUCTION

Menopause is defined as the permanent cessation of menstruation resulting from a natural decline in estrogen and progesterone and typically occurs around the age of 50^1^. The age at onset varies according to genetic, environmental, socioeconomic, and cultural factors^2^. A multinational study involving over 500,000 women from 10 countries reported a mean age of menopause of 50.5 years^3^. In contrast, studies conducted among Turkish women indicate that the average age at menopause ranges from 47 to 51 years^4^.

Hypoestrogenism during menopause leads to physical, psychological, and sexual symptoms, including vasomotor symptoms, sleep disturbances, decreased libido, vaginal dryness, anxiety, fatigue, and headaches^2,4^. Approximately 80% of women experience vasomotor symptoms such as hot flashes and night sweats^5^. Menopause is also associated with an increased risk of depression and anxiety, negatively affecting quality of life and daily functioning^6,7^. These psychological symptoms may be partly explained by hypoestrogenism-related alterations in neurotransmitter metabolism, which increase women's vulnerability to depression^8^.

A meta-analysis involving 385,092 postmenopausal women reported a depression prevalence of 28%^7^. Compared with premenopausal women, both depression and anxiety disorders are more common during the postmenopausal period^9^. Due to concerns regarding the risks of Menopausal Hormone Therapy (MHT), many women seek complementary and alternative approaches^10^.

One such approach is the Emotional Freedom Technique (EFT), a mind–body intervention that combines cognitive techniques with acupressure-based somatic stimulation. The technique involves focusing on a distressing thought or emotion while applying somatic stimulation to 12 specific meridian points. Evidence suggests that EFT provides beneficial psychological and physiological effects^11,12^. EFT involves tapping on specific acupuncture points while verbalizing affirmations related to emotional distress^13^. Previous studies have demonstrated its effectiveness in reducing anxiety, depression, premenstrual symptoms, and treatment-related menopausal complaints in different populations^14,15^.

To date, only one study has evaluated EFT in menopausal women, reporting that an 8-week intervention significantly reduced depressive symptoms^16^. Therefore, the present study aimed to evaluate the effects of EFT on depressive and menopausal symptoms in postmenopausal women.

## METHODS

### Protocol

The study was approved by the Hitit University Non-Interventional Research Ethics Committee (02.12.2022; No: 2022-26) and registered on ClinicalTrials.gov (NCT06338085). The study was reported in accordance with Consolidated Standards of Reporting Trials guidelines.

### Participants

The study was conducted between January and July 2023 in a single family health center in Central Anatolia, Turkey, and included 70 volunteer postmenopausal women. Participants were recruited continuously throughout the study period, and both groups were enrolled concurrently, thereby minimizing potential seasonal effects. Verbal and written informed consent was obtained from all participants, and the study was conducted in accordance with the Declaration of Helsinki.

Inclusion criteria were literacy, postmenopausal status, ability to communicate effectively, and provision of consent. Exclusion criteria included surgical menopause, clinically diagnosed psychiatric illness, and receipt of MHT. Participants receiving hormone therapy were excluded to minimize potential confounding effects. Use of antidepressant medication or herbal supplements was assessed at baseline as part of participant characteristics. Exclusion criteria did not include Beck Depression Inventory (BDI) scores, which were used solely as a screening tool to assess depressive symptom severity and not for diagnostic or exclusion purposes. Therefore, participants with elevated BDI scores but without a formal psychiatric diagnosis were included in the study.

### Randomization and blinding

Postmenopausal women who met the inclusion criteria were assigned to study groups using a computer-assisted simple randomization method. The random allocation sequence was generated by an independent researcher not involved in the study, using Random.org (a random integer generator). In the allocation sequence, code 1 represented the EFT group and code 2 represented the control group. Participants were sequentially numbered according to their order of enrollment and assigned to study groups based on the pre-generated randomization list. The allocation sequence was concealed from the researcher responsible for participant assignment. Blinding was not feasible due to the nature of the intervention and the involvement of the researcher in both intervention delivery and outcome assessment.

### Sample size

Sample size estimation was based on effect sizes reported in previous similar mind-body intervention studies in the literature. Using G*Power (version 3.1.9.7), a priori power analysis was conducted with a two-tailed hypothesis, 80% power (1-β) and 95% confidence level (1-α). Based on an effect size of d=0.69 derived from previous studies, a minimum of 34 participants per group (total n=68) was required. To account for an anticipated 10% attrition rate, 76 women (38 per group) were initially enrolled. During the study, four participants in the EFT group and two participants in the control group withdrew. Therefore, the final sample consisted of 70 women (EFT: n=34; control: n=36). Post-hoc power analysis indicated a statistical power of 0.98 for the observed effect size (d=0.99). This analysis was reported for descriptive purposes only and not used for the original sample size determination.

### Intervention

All researchers held EFT practitioner certificates. The EFT intervention was delivered by third researcher Meryem VURAL ŞAHİN in accordance with standard EFT principles and guidelines.

### Emotional Freedom Technique group

**Figure 1:**
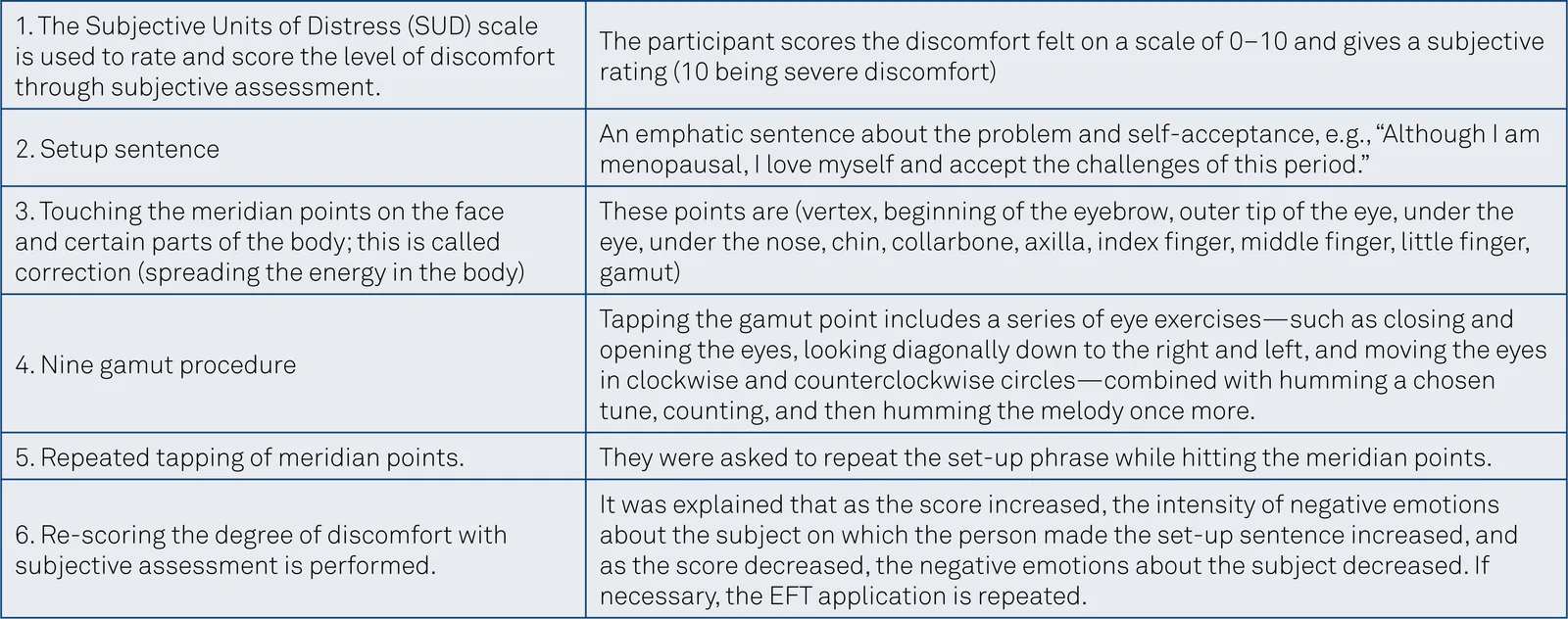
Emotional Freedom Technique implementation steps^17^.

Participants attended a single face-to-face introductory session during which EFT points and application steps were explained, and a full session was demonstrated. An educational video, prepared according to EFT guidelines, was shared via WhatsApp. Participants were instructed to perform EFT at least once daily for eight weeks, with adherence supported through daily WhatsApp reminders and weekly follow-up phone calls. During weekly calls, participants provided self-reports regarding their practice. However, adherence was not recorded using a structured log or objective monitoring system; therefore, the exact frequency, duration of sessions, and total number of completed EFT practices could not be quantified. In addition, intervention fidelity (i.e., whether EFT was performed correctly according to protocol steps) was not formally assessed. The intervention process is shown in Figure 1. Participants assigned to the EFT group were instructed not to use additional interventions that could influence menopausal symptoms, including herbal supplements and exercise, during the study period.

### Control group

The control group received routine care, including standard health consultations and educational materials on menopausal health, and after the follow-up period, they were provided with EFT training and access to training videos in accordance with ethical principles.

### Measurements

All assessments were conducted by the researcher at baseline and at the end of the eighth week. At baseline, participants completed a sociodemographic form, the Menopause Rating Scale (MRS), and the BDI. All measurements were repeated for both groups following completion of the intervention.

### Outcomes

The outcomes of this study were changes in menopausal symptoms and depressive symptoms among postmenopausal women. Both outcomes were evaluated to determine the effect of EFT on symptom severity.

In this study, the MRS was used to assess menopausal symptoms. The scale, originally developed by Schneider et al., has been validated and shown to be reliable. The Turkish validity and reliability of the MRS were established by Gürkan^18,19^. The MRS consists of 11 items covering somatic, psychological, and urogenital symptoms, scored on a 0–4 Likert scale, with total scores ranging from 0 to 44. Higher scores indicate greater symptom severity. In this study, Cronbach's alpha coefficients were 0.90 for the EFT group and 0.91 for the control group.

Depressive symptoms were evaluated using the BDI, a 21-item self-report scale validated for Turkish populations^20^. Items are scored from 0 to 3, yielding total scores between 0 and 63, with higher scores reflecting greater symptom severity. Cronbach's alpha values in the present study were 0.94 for the EFT group and 0.93 for the control group.

### Statistical analysis

Data were analyzed using Statistical Package for the Social Sciences *(*SPSS) version 25.0 for Windows (SPSS Inc., Chicago, IL, USA). Data normality was assessed using the Kolmogorov-Smirnov test. Descriptive statistics were presented as counts, percentages, mean±standard deviation, and minimum–maximum values. Group comparisons were performed using the Independent Samples t-test, Mann-Whitney U test, and chi-square test, with p<0.05 considered statistically significant.

## RESULTS

**Figure 2:**
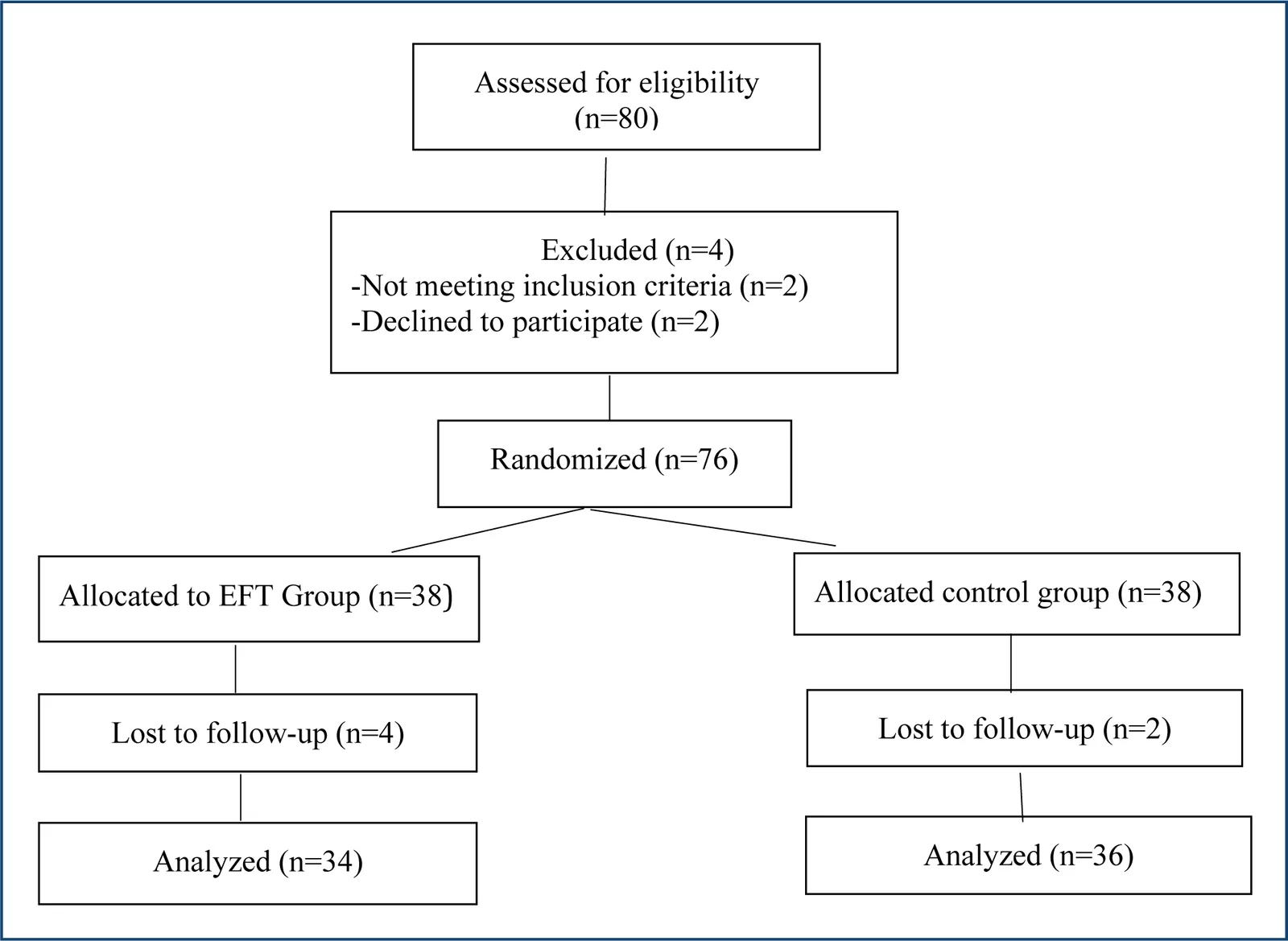
A flow chart of the study groups.

The study sample consisted of 70 postmenopausal women (EFT group: 34; control group: 36) (Figure 2). Sociodemographic characteristics, including age, body mass index, gravida, parity, duration of menopause, education level, income, employment status, marital status, smoking status, and chronic disease status, were similar between the groups (p>0.05) (Table 1).

**Table 1:** Demographic characteristics of participants.

Quantitative variables	Control group n=36			EFT group n=34			p
	$\bar{X}$ ±SD	$\bar{X}$	Min–max	$\bar{X}$ ±SD	$\bar{X}$	Min–max	
Age, years^a^	54.42±6.78	53.50	43–69	53.06±5.33	53.00	45–65	0.357
Height^a^	1.61±0.06	1.62	1.50–1.70	1.62±0.05	1.62	1.50–1.70	0.270
Weight^a^	69.94±9.56	70.00	52–105	71.26±8.62	70.50	54–100	0.547
BMI^b^	27.06±3.67	27.24	21.0–41.0	27.05±2.88	26.73	21.1–41.0	0.810
Gravida^b^	2.25±1.30	2.00	0–7	2.47±1.13	2.00	0–5	0.261
Parity^b^	1.86±0.93	2.00	0–4	2.00±0.82	2.00	0–4	0.521
Menopause duration^b^	6.72±5.90	4.00	1–23	5.56±3.71	4.50	1–12	0.799
**Qualitative variable**		**n**	**%**		**n**	**%**	
Education level^b^	Literate	1	2.8	Literate	0	0.0	0.845
	Primary school	5	13.9	Primary school	7	20.6	
	Middle school	2	5.6	Middle school	1	2.9	
	High school	18	50.0	High school	15	44.1	
	University	10	27.8	University	11	32.4	
Income level^b^	Poor	8	22.2	Poor	3	8.8	0.224
	Average	22	61.1	Average	24	70.6	
	Good	6	16.7	Good	7	20.6	
Working status^c^	Working	8	22.2	Working	9	26.5	0.598
	Retired	10	27.8	Retired	6	17.6	
	Not working	18	50.0	Not working	19	55.9	
Marital status^c^	Married	28	77.8	Married	31	91.2	0.124
	Single	8	22.2	Single	3	8.8	
Smoking^c^	Never smokers	25	69.4	Never smokers	19	55.9	0.241
	Current smokers	11	30.6	Current smokers	15	44.1	
Chronic disease^c^	With disease	18	50.0	With disease	17	50.0	1.000
	Without disease	18	50.0	Without disease	17	50.0	

a Independent two samples t-test;

b Mann-Whitney U test;

c Chi-square independence test. EFT: Emotional Freedom Technique; BMI: body mass index; SD: standard deviation.

As shown in Table 2, no significant differences were found between the groups in baseline MRS total or subdimension scores (p>0.05). However, post-test analyses demonstrated significant improvements in the EFT group compared with the control group for MRS total scores and somatic (p<0.001), psychological (p<0.001), and urogenital (p=0.005) subdimensions.

**Table 2:** Mean of menopausal symptoms and depression score in two groups of intervention and control.

		Control group n=36	EFT group n=34	Between-groups p-value
Total MRS	Pre-test	17.44±8.64	17.79±7.08	0.854
	Post-test	17.39±8.20	9.56±6.12	<0.001
	Within group p-value	0.970	<0.001	
Sub-dimensions of MRS				
Somatic	Pre-test	2.61±1.79	2.59±1.56	0.955
	Post-test	2.75±1.59	1.24±1.02	<0.001
	Within group p-value	0.664	<0.001	
Psychological	Pre-test	10.42±4.74	10.97±5.02	0.637
	Post-test	10.39±5.08	6.03±4.01	<0.001
	Within group p-value	0.977	<0.001	
Urogenital	Pre-test	4.42±3.08	4.24±1.62	0.757
	Post-test	1.58±1.65	0.65±0.98	0.005
	Within group p-value	<0.001	<0.001	
Total BDI score	Pre-test	15.69±10.82	15.18±11.56	0.847
	Post-test	14.86±11.55	5.65±6.18	<0.001
	Within-group p-value	0.709	<0.001	

Note: Mean±standard deviation values are given in the table. EFT: Emotional Freedom Technique; MRS: Menopause Rating Scale; BDI: Beck Depression Inventory.

Table 2 presents BDI results. Baseline BDI scores were comparable between groups (p=0.847), whereas post-test scores were significantly lower in the EFT group (p<0.001). Following the intervention, the EFT group exhibited marked reductions in symptom severity, including a 62.78% decrease in BDI scores and a 46.26% decrease in MRS total scores, alongside improvements across all MRS subdimensions. The EFT group indicated a significant reduction in BDI scores (p<0.001, Cohen's d=1.02). MRS scores also decreased significantly in this group (p<0.001, Cohen's d=1.25).

## DISCUSSION

Although previous studies have examined EFT in various populations, few have simultaneously investigated its effects on both depressive and menopausal symptoms in postmenopausal women. This study provides novel evidence demonstrating the effectiveness of EFT as a non-pharmacological intervention for managing these symptoms. Furthermore, these findings highlight the potential for integrating EFT into routine clinical care as an innovative and effective intervention for postmenopausal women.

Studies indicate that the physiological and psychological changes occurring during the postmenopausal period intensify menopausal symptoms and increase the risk of depression in a substantial proportion of women^7,21^. The coexistence of menopausal symptoms and psychological problems adversely affects quality of life and overall well-being^22^. Due to concerns about the risks associated with MHT, many women prefer alternative approaches for symptom management^10^. In this context, EFT may serve as an easily applicable method for reducing depressive and menopausal symptoms in postmenopausal women.

In the present study, there were no significant differences between the EFT and control groups in terms of age, body mass index, obstetric characteristics, duration of menopause, or sociodemographic variables, indicating baseline comparability between groups (Table 1).

Our findings showed that BDI scores in the EFT group decreased significantly after the intervention compared with pre-test values (p<0.001). While no significant differences were observed between the EFT and control groups at baseline, post-test BDI scores were significantly lower in the EFT group. These results indicate that EFT was effective in reducing the severity of depressive symptoms.

In the literature, only one study has examined the effects of EFT on depressive symptoms in postmenopausal women, reporting a substantial reduction in BDI scores following the intervention^16^. Consistent with this finding, our study demonstrated a 62.78% decrease in BDI scores in the EFT group. Similar benefits have been observed with other psychological interventions. For example, a 24-week cognitive behavioral therapy program resulted in improvements in both vasomotor symptoms and recurrent depressive symptoms among postmenopausal women^23^.

Previous research across diverse populations has similarly shown that EFT effectively reduces depression and anxiety symptoms^12,14,15,24^. Meta-analyses further support the high efficacy of EFT in alleviating depressive symptoms, with effect sizes comparable to or exceeding those reported for conventional psychotherapeutic and pharmacological interventions^12,15^. The present study adds to the existing literature by confirming the beneficial effects of EFT on depressive symptoms in postmenopausal women.

Previous studies report that postmenopausal women frequently experience symptoms such as hot flashes, night sweats, sleep disturbances, fatigue, and headaches, with meta-analytic evidence showing an increased prevalence of these symptoms during the postmenopausal period^25–27^. Furthermore, anxiety, depression, and stress are emotional states that may contribute to the development and maintenance of physical symptoms and pain, emphasizing the close relationship between psychological well-being and symptom burden^28^. In the present study, total MRS scores and all sub-dimensions (somatic, psychological, and urogenital) in the EFT group decreased significantly after the intervention (p<0.001). While no significant differences were observed between groups at baseline, post-test MRS total and sub-dimension scores were significantly lower in the EFT group (p<0.05). Overall, EFT led to marked reductions in menopausal symptom severity, supporting its effectiveness as a non-pharmacological intervention.

Although no previous studies have directly examined the effects of EFT on menopausal symptoms in postmenopausal women, findings from related research support its potential benefits. A study among cancer survivors reported significant reductions in depression, anxiety, and fatigue, along with improvements in quality-of-life following EFT^29^. Consistent with these findings, the present study suggests that EFT enhances women's ability to cope with menopausal symptoms, leading to a reduction in symptom severity.

In conclusion, EFT sessions in postmenopausal women were effective in alleviating depressive symptoms and menopausal complaints. These results indicate that EFT may serve as a beneficial non-pharmacological approach for supporting mental health and managing menopausal symptoms in this population.

### Limitations and strengths

This study is limited by its single-center design and relatively small sample size, which may limit generalizability. Blinding was not feasible due to the nature of the intervention, which may have introduced performance and detection bias. As outcomes were self-reported (BDI and MRS), results may also be influenced by expectation bias. However, the statistician was blinded to group allocation to reduce analytical bias.

In addition, the control group received routine care and educational materials, whereas the EFT group received additional attention through daily reminders and weekly follow-up calls. Therefore, observed differences may partly reflect non-specific effects rather than EFT-specific effects. The control condition was not matched for contact time, and future studies should consider attention-control conditions such as sham tapping or equivalent follow-up support.

Finally, adherence to EFT was not systematically monitored, and intervention fidelity was not assessed; therefore, the exact intervention dose could not be determined. Future research should incorporate standardized adherence and fidelity measures and examine long-term outcomes. This is the first study to evaluate the effects of EFT on menopausal symptoms in postmenopausal women.

## Data Availability

The datasets generated and/or analyzed during the current study are available from the corresponding author upon reasonable request.
